# Feldspar flotation as a quartz-purification method in cosmogenic nuclide dating: A case study of fluvial sediments from the Pamir

**DOI:** 10.1016/j.mex.2018.06.014

**Published:** 2018-06-28

**Authors:** Vasila A. Sulaymonova, Margret C. Fuchs, Richard Gloaguen, Robert Möckel, Silke Merchel, Martin Rudolph, Matthias R. Krbetschek

**Affiliations:** a Geologie, TU Bergakademie Freiberg, Germany; b University of Central Asia, Kyrgyzstan; c Applied Physics, TU Bergakademie Freiberg, Germany; d Helmholtz-Zentrum Dresden-Rossendorf, Helmholtz Institute Freiberg for Resource Technology, Germany

**Keywords:** Froth flotation, Mineral separation, Quartz, Feldspar, Accelerator mass spectrometry, Cosmogenic nuclide dating

## Abstract

Cosmogenic nuclide (CN) dating relies on specific target minerals such as quartz as markers to identify geologic events, including the timing of landscape evolution. The presence of feldspar in sediment samples poses a challenge to the separation of quartz and affects the chemical procedures for extracting the radioactive CNs ^10^Be and ^26^Al. Additionally, feldspar contamination reduces the ^26^Al/^27^Al ratio, thus hinders the accurate determination of ^26^Al by accelerator mass spectrometry (AMS). Using fluvial sediment samples from Central Asia, which contain 16–50 weight percent (wt.%) of feldspar, we show that the standard physical separation and chemical cleaning-up procedures for quartz-enrichment reduces the feldspar content to only 9–47 wt.%. We present a new froth flotation mineral-separation device and procedure that allows for very effective quartz enrichment before CN chemistry. Our flotation cell, which has a volume of 600 cm^3^, is built of borosilicate glass, holds up to 90 g of sample, and achieves quartz and feldspar separation in ≤2 h for very feldspar-rich samples. We trace the stepwise enrichment of quartz to 95–100% purity with our procedure by X-ray diffraction analysis.

## Highlights

• Our flotation cell is built of borosilicate glass, holds up to 90 g of sample, and achieves quartz and feldspar separation in ≤2 h.
• The procedure uses air bubbles to which the feldspars attach, 0.2% HF to reduce the surface energy of quartz, dodecylamine solution as a feldspar collector, and operates at a pH range of 2.4–2.7 at room temperature.
• We trace the stepwise enrichment of quartz by X-ray diffraction analysis, which shows that froth flotation is the decisive step to reach 95–100% purity from the initial 23–46%.

## Graphical abstract

**Figure:**
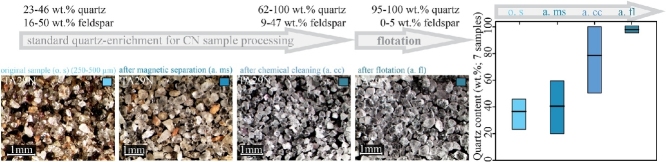

**Specifications Table**

**Table:** 

Subject area	• *Earth and Planetary Sciences*
More specific subject area	*Mineral Processing*
Method name	*Froth flotation*

## Introduction

The *in-situ* produced cosmogenic nuclides (CN) ^10^Be (t_1/2_ = 1.387 Ma) and ^26^Al (t_1/2_ = 0.705 Ma) [[1], [2], [3]] are commonly extracted from quartz because the nuclear reactions leading to the production of these nuclides are well-understood. Their concentrations are extremely low (10^4^–10^9^ atoms/g), which requires measurements by accelerator mass spectrometry (AMS; [[4], [5], [6]]). Standard sample preparation in CN applications comprises physical quartz-enrichment, chemical cleaning, Be and Al separation, BeO and Al_2_O_3_ production, and target preparation. The physical quartz-enrichment is crucial for ensuring an effective chemical cleaning with a mixture of HCl and H_2_SiF_6_ [7] before the extraction of CN from quartz. High non-quartz components after physical cleaning reduce the quality of chemical cleaning and a high proportion of chemicals will be consumed before all non-quartz minerals are dissolved. The quartz cleaning may be improved by applying multiple HCl/H_2_SiF_6_ chemical cleaning cycles. But this is payed by increased economic and ecologic costs, and also by a continuous reduction of the quartz fraction due to rinsing losses. Furthermore, for our samples, also repeated chemical cleaning for up to 6 cycles did not guarantee pure quartz extracts and thereby, would introduce uncertainties in the consecutive CN extraction if not addressed properly. Therefore, we focus on an efficient physical quartz-enrichtment. Classically, this involves sieving, ultra-sonic bath, and magnetic and density separation. However, these sample preparation steps were insufficient for our river and fluvial terrace samples from the Pamir, Central Asia, which contain feldspar concentrations as high as 16–50 weight percent (wt.%), typical for samples from eroding active mountain belts. The sediment source areas of our samples comprise high-grade metamorphic, igneous, and weakly to non-metamorphic source rocks (e.g., [[8], [9], [10], [11]]). To overcome the obstacles of non-sufficient physical quartz-enrichment motivated us to investigate further options for separating quartz from feldspar.

Separating quartz from feldspar is difficult due to similar densities and magnetic susceptibilities. A useful separation method for both minerals is froth flotation [12,13], which combines physical and chemical treatments, and is known as an effective method in the mining industry for the beneficiation of feldspar.’ Feldspar’ describes a group of framework silicates, mainly containing Si, Al, Ca, K, and Na but may also include Ba, Ti, Fe, Mg, Sr, and subsidiary Mn [[13], [14], [15]]. Our samples from Central Asia contain both plagioclase (Pl) and K-feldspar (K-fsp).

This study describes a modified glass flotation cell for the separation of quartz from feldspar with grain sizes of 250–1000 μm. Fuerstenau et al. [16] and Arnold et al. [17] introduced a glass flotation cell, the Hallimond tube, to test the effects of reagent-concentration variations on froth flotation used in the industry. Their flotation cell operates at a mini-pilot scale, allowing the processing of up to 3 g of sample material. Clifton et al. [18] and Gibbon et al. [19] introduced froth flotation for mineral separation in CN applications. In contrast to our work (see Section Froth flotation: new cell and operation procedure), they used CO_2_ bubbles to separate feldspar from quartz. From our experience and personal communication, we learned that froth flotation is not a standard procedure for CN sample preparation, usually because samples by chance had much lower feldspar concentrations. In case of increased feldspar concentrations, alternative solutions such as using soda-carbonators and adding e.g. eucalyptus oil did not produce comparably pure quartz extracts. The visits to other labs and a test of another flotation cell “GTK LabCell” confirmed that especially the adjustment of air pressure for a smooth bubble generation and handling of froth on top of the solution is of concern. We document the effectiveness of our approach by providing the mineral content in Table 1.

**Table 1:** Samples location and their mineralogical compositions from X-ray diffraction analysis.

	Sample name	**TA18N**	**TA19N**	**TA17O**	**TA17N**	**TA01E**	**TA29N**	**TA05O**
	**Location**	Shaymak	Shaymak	Kona Kurgan	Murgab	Yazgulom	Patkhur	Batshor south
	**Latitude [°N]**	37.502	37.461	38.184	38.155	38.192	37,713	37.750
	**Longitude [°E]**	74.835	74.823	76.066	73.968	71.372	72.208	72.441
	**Altitude** [m a.s.l.]	3867	3861	3636	3601	2368	3047	3259

**(1) Original sieved sample (wt.%)**	Mica	10.6 ± 0.2	11.5 ± 0.4	5.0 ± 0.2	9.8 ± 0.3	7.8 ± 0.2	5.9 ± 0.3	11.9 ± 0.2
	Calcite	19.4 ± 0.1	2.4 ± 0.1	27.2 ± 0.2	23.9 ± 0.1	33.3 ± 0.2	0	0
	Quartz	28.1 ± 0.2	45.5 ± 0.2	23.1 ± 0.2	36.8 ± 0.2	33.8 ± 0.2	42.5 ± 0.1	45.8 ± 0.2
	Plagioclase	19.2 ± 0.2	21.1 ± 0.2	16.2 ± 0.2	18.8 ± 0.2	12.3 ± 0.2	32.1 ± 0.7	24.2 ± 0.1
	Chlorite	7.0 ± 0.2	3.3 ± 0.2	1.8 ± 0.2	2.5 ± 0.1	1.9 ± 0.2	<1	<1
	Amphibole	0	<1	5.0 ± 0.1	1.5 ± 0.1	1.4 ± 0.1	1.1 ± 0.1	<1
	Dolomite	6.2 ± 0.1	0	18.6 ± 0.2	1.2 ± 0.1	5.6 ± 0.1	0	0
	K-Feldspar	9.4 ± 0.3	15.6 ± 0.6	3.2 ± 0.2	5.5 ± 0.2	3.9 ± 0.2	17.9 ± 0.4	16.7 ± 0.8

**(2) Aftermagnetic separation (wt.%)**	Mica	<1	<1	<1	1.6 ± 0.3	1.5 ± 0.2	1.7 ± 0.1	2.1 ± 0.2
	Calcite	50.9 ± 0.2	3.3 ± 0.1	52.5 ± 0.2	22.3 ± 0.1	44.8 ± 0.2	0	0
	Quartz	20.1 ± 0.2	52.2 ± 0.3	24.1 ± 0.2	45.0 ± 0.2	36.7 ± 0.2	46.6 ± 0.2	59.5 ± 0.3
	Plagioclase	11.8 ± 0.2	31.5 ± 0.3	15.0 ± 0.2	18.7 ± 0.3	11.5 ± 0.2	28.5 ± 0.3	17.0 ± 0.3
	Chlorite	0	0	0	0	0	0	0
	Amphibole	0	0	0	0	0	0	0
	Dolomite	7.3 ± 0.2	0	2.2 ± 0.1	0	2.0 ± 0.1	0	0
	K- Feldspar	9.9 ± 0.3	12.5 ± 0.3	5.3 ± 0.4	12.3	3.5 ± 0.3	19.2 ± 0.3	21.4 ± 0.7

**(3) After chemical cleaning (wt.%)**	Mica	<1	0	0	2.4 ± 0.2	0	0	2.2 ± 0.1
	Calcite	0	<1	0	0	0	<1	<1
	Quartz	80.7 ± 0.3	82.7 ± 0.3	91.4 ± 0.2	82.9 ± 0.3	100.0	62.1 ± 0.3	50.5 ± 0.2
	Plagioclase	11.7 ± 0.3	11.1 ± 0.2	8.6 ± 0.2	9.5 ± 0.2	0	19.1 ± 0.3	25.1 ± 0.3
	Chlorite	0	0	0	0	0	0	0
	Amphibole	0	0	0	0	0	0	0
	Dolomite	0	0	0	0	0	0	0
	K- Feldspar	7.1 ± 0.3	6.0 ± 0.2	0	5.3 ± 0.2	0	17.9 ± 0.3	22.3 ± 0.6

**(4) After flotation (wt.%)**	Mica	0	0	0	1.2 ± 0.2	0	0	0
	Calcite	0	0	0	0	0	0	0
	Quartz	97.8 ± 0.1	95.6 ± 0.2	100.0	95.5 ± 0.2	100.0	97.2 ± 0.1	95.1 ± 0.2
	Plagioclase	2.2 ± 0.2	4.4 ± 0.2	0.0	3.3 ± 0.2	0	2.5 ± 0.1	3.6 ± 0.1
	Chlorite	0	0	0	0	0	0	0
	Amphibole	0	0	0	0	0	0	0
	Dolomite	0	0	0	0	0	0	0
	K- Feldspar	0	0	0	0	0	<1	1.3 ± 0.2

Within this study, we present our alternative froth flotation that takes advantage of a tunable air pump for full control of bubble generation and reduces the loss of quartz during processing. We emphasize the benefits regarding costs in time and money as well as safety, and that it can easily be applied to other geochronological methods. For example, we [20] used the flotation cell as the routine quartz and feldspar separation method for fluvial terrace dating by optically stimulated luminescence (OSL). Herein, we demonstrate the efficiency of the feldspar-flotation method using seven fluvial sediment samples targeted for CN dating. We quantified the purity of the quartz achieved after the various enrichment steps using X-ray diffraction (XRD) analysis.

## Materials and methods

### Sampling sites

**Fig. 1:**
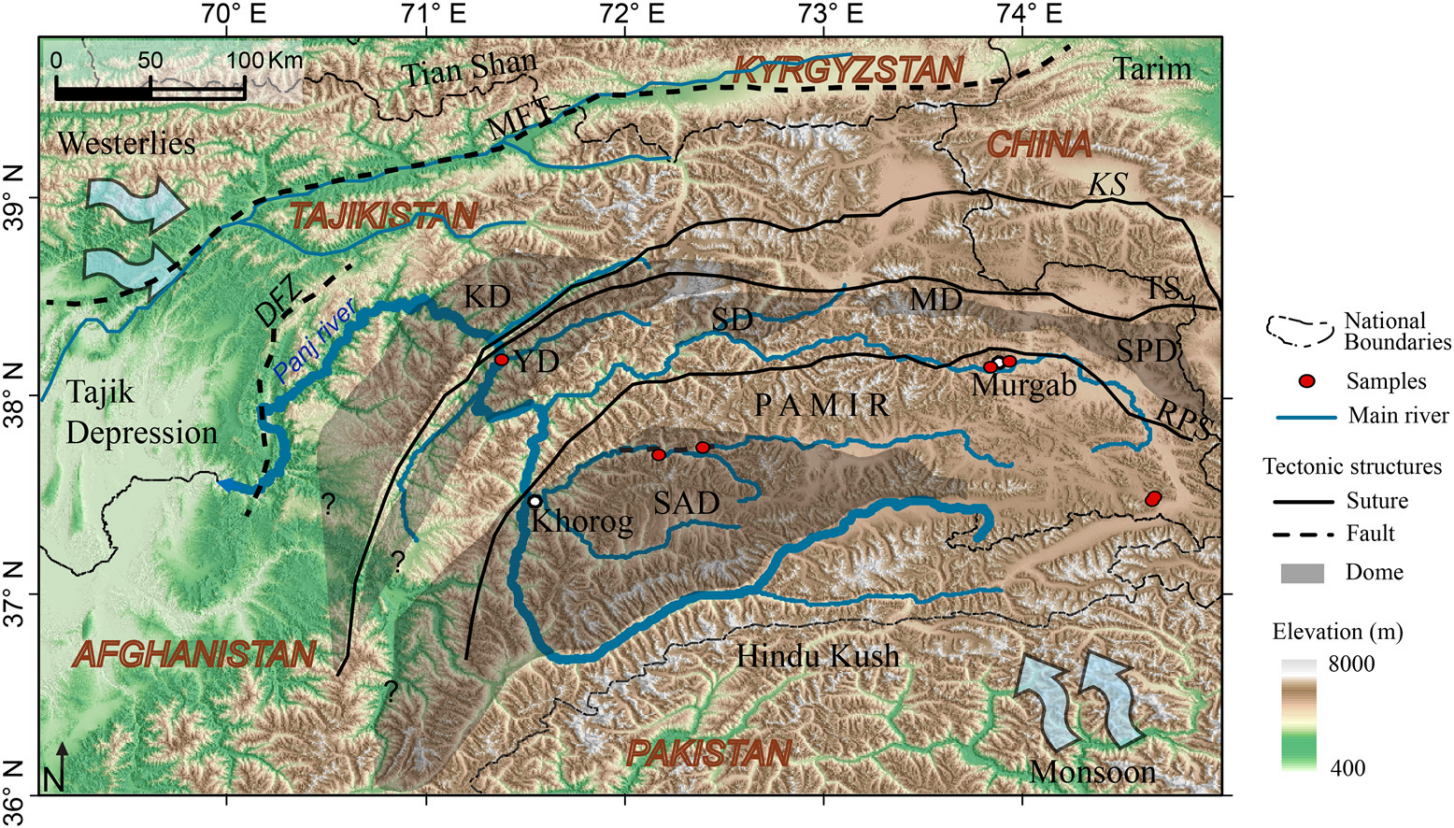
Map of the Pamir with the Panj River drainage. Atmospheric circulations, geologic structures, and sample localities are marked. Abbreviations: DFZ, Darvaz Fault Zone; MFT, Main Frontal Thrust; KS, Kunlun Suture; TS, Tanymas Suture; RPS, Rushan-Pshart Suture; KD, Kurgovat Dome; MD, Muskol Dome; SPD, Shatput Dome; YD, Yazgulom Dome; SD, Sarez Dome; SAD, Shakhdara-Alichur Dome.

The Pamir of Central Asia resulted from the Cenozoic Indian–Asian collision, which induced changes in the relief and the drainage system [21]. The unique position of the Pamir between two atmospheric circulations - the Westerlies and Indian Summer Monsoon - and its ongoing tectonic activity at the northwestern tip of the India-Asia collision, make the region attractive for CN dating applications. The material used herein comprises fluvial sediments from the major rivers draining the Pamir (Fig. 1; Table 1). The sediments transported and deposited in those rivers have integrated the erosion products from the various rocks exposed in Pamir. These rocks are part of micro-continents, subduction-accretion complexes, and magmatic arc terranes that assembled during the Paleozoic and Mesozoic (e.g. [8,22]); by their nature, their lithology is variegated. The highest denudation rates in the Pamir have been determined in Cenozoic gneiss domes that cover about 30% of the Pamir (e.g. [[9], [10], [11],[23], [24], [25]]). They contain high-grade metamorphic rocks of sedimentary, volcanic, and plutonic protoliths, and ultramafic to felsic igneous rocks. The widespread high-grade metamorphic and igneous rocks delivered high amounts of feldspar into the river sediments.

### Sample preparation

**Fig. 2:**
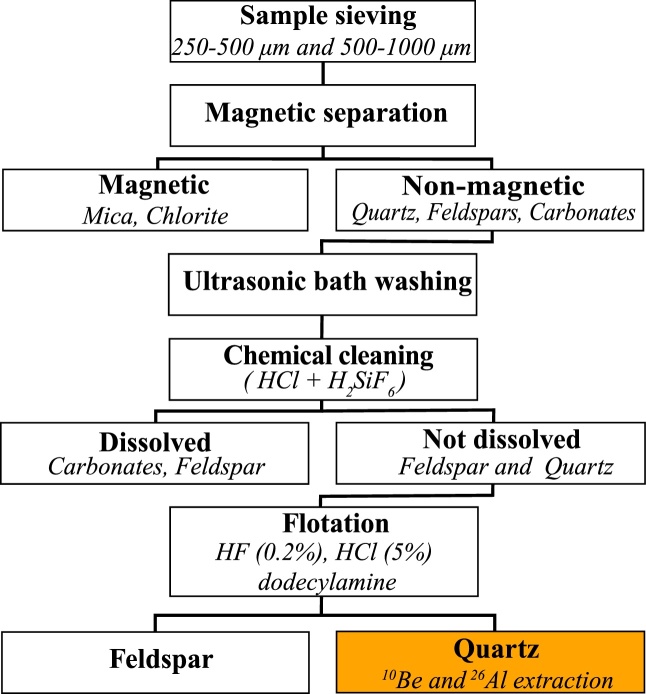
Flow diagram showing the stepwise procedure for separation quartz from river-sand samples.

Fig. 2 shows a flow diagram of the sample preparation procedure. The samples were crushed and wet sieved into 250–500 μm and 500–1000 μm grain-size fractions. After drying, representative subsamples were taken for XRD analysis. We first separated the strongly magnetic minerals using a hand magnet, and then used a Frantz magnetic separator to isolate mica and chlorite. Again, subsamples were taken for XRD analysis. A 1:1 solution of HCl (32%) and H_2_SiF_6_ (34%) was then used to dissolve the carbonates, feldspar, and organic material [7]. The samples were shaken horizontally in 250 ml polyethylene bottles at room temperature overnight. The supernatants were decanted and the samples rinsed with deionized water until clear. After applying six acid wash cycles, the XRD results demonstrated that the samples still contained ∼9–47% feldspar (Table 1, after the chemical cleaning step; both Pl and K-fsp). As stated above, procedures for quartz-enrichment in CN applications also suggest density separation, which proved successful for the separation of various mineral phases. However, due to the similar densities of quartz and feldspar [26], density separation was unsuccessful for reducing the feldspar in our samples. Here, flotation provides a solution, as it takes advantage of the different wetting characteristics and surface properties of quartz and feldspar [12,[27], [28], [29]].

### Froth flotation: new cell and operation procedure

**Fig. 3:**
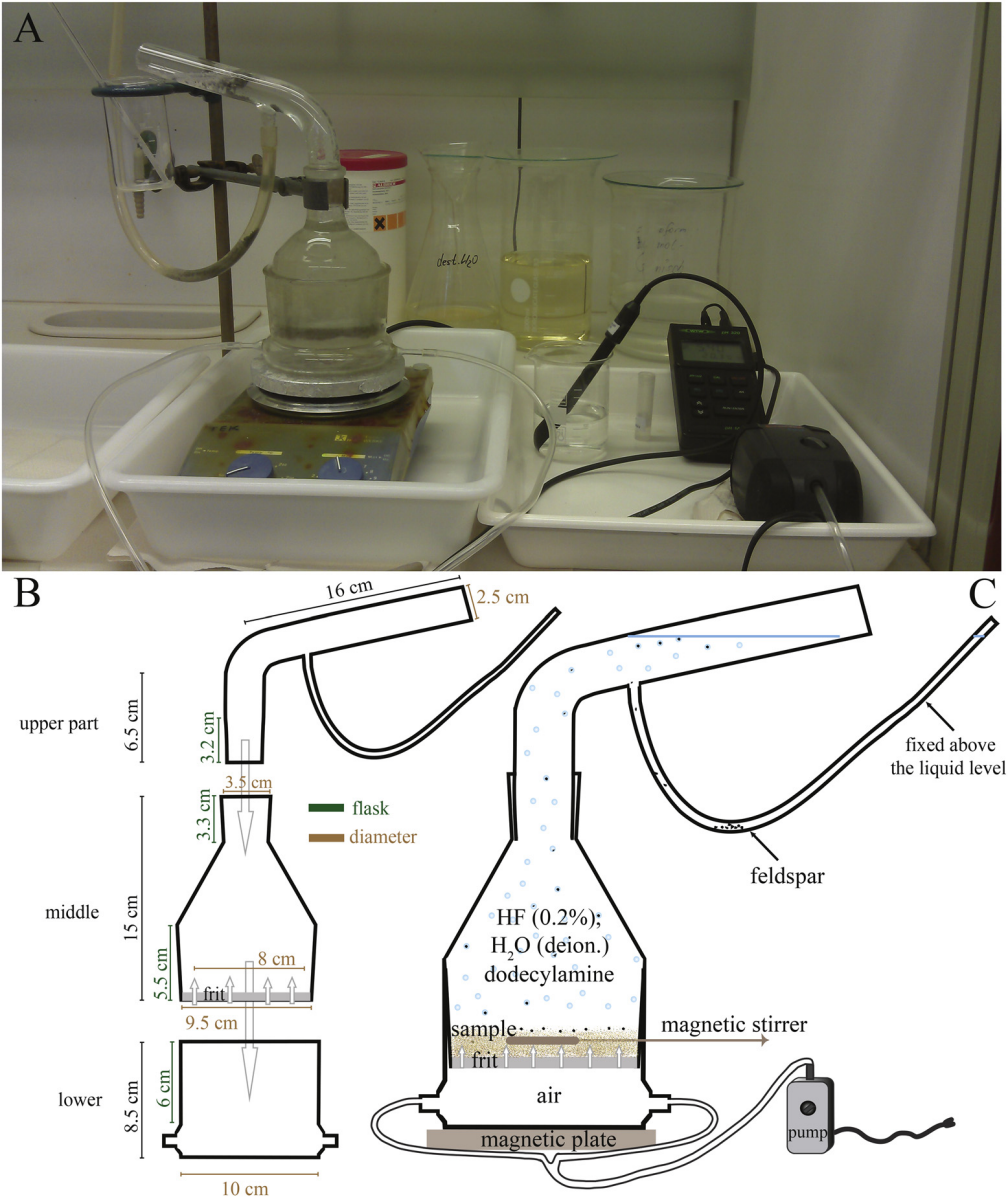
Flotation-cell design. **(A)** Photo of the flotation cell during operation; the devices on the right side are the pH meter and the air pump. **(B)** The three parts of the flotation cell with their dimensions. **(C)** The assembled flotation cell under operation.

Our modified flotation cell consists of three glass parts manufactured in a Laboratory Supply Shop (Fig. 3A). The lower part, in total 8.5 cm high and 10 cm in diameter, has two 0.6 cm diameter holes for the tubes that introduce air via an air pump; the part above the air inlets is 6 cm high (Fig. 3B). The 16 cm high middle part is 9.5 cm in diameter and fits tightly into the lower part, preventing leakage of chemicals/sample (see below). It narrows upward to the top flask part with a 3.5 cm diameter. This middle part has an 8 cm diameter ceramic frit glued into its open lower part. The ceramic frit is 0.6 cm thick, has porosity 2 (according to “ISO identification mark P100”), and nominal maximum pore size 40–100 μm allows the passage of the air and the generation of bubbles by dispersion in the liquid-sample mixture during operation (see explanation below). The upper part is 24 cm long and 2.5 cm in diameter; the lower 3.2 cm fit tightly into the top of the middle part, again preventing leakage of liquid (see operation part below). Above this lower section, the upper part bends, forming a tube that is inclined 15° from horizontal; its top end is open. At the lower end of its outward-bent snout, a hole with an attached flexible tube of 1 cm diameter allows the collection of the feldspar (see below; Fig. 3A and B). We used compressed air from an air pump for bubble generation. As the type of pump and the generated pressure is not important, we used a commercially available and cheap pump, which is generally used for aquariums. It allows the adjustment of the airflow to permit a fine-tuning of the bubble generation. Given the specifications, the calculated pressure is ∼0.00443 Pa. The entire flotation cell rests on the magnetic plate allowing sample-liquid mixing via a stirrer (see below; Fig. 3A–C).

During operation, the air pump is started first. It introduces air through the tubes into the lower part of the cell, from where it rises through the ceramic frit to the middle and upper parts of the cell. Next, the middle part of the flotation cell is filled with up to 90 g of sample material, previously wetted with HF (0.2%). The HF reduces the surface energy of the quartz [27]. The rising air prevents leakage of the liquid-sample mixture through the frit. As a collector for feldspar, we add 6 ml of a dodecylamine solution to the HF-sample mixture. The solution is prepared from dry powder dodecylamine (98%) in proportion of 1 g dodecylamine (98%) + 5.4 ml HCl (1 N) + 100 ml H_2_O (deionized). Then, the HF-sample is covered with additional HF (0.2%) at a pH range of 2.4–2.7 to above the opening of the flexible tube; the latter is fixed at a level higher than the fluid level to prevent spilling of the HF. For the adjustment of the pH value to the desired range, H_2_SO_4_ (1 N) or deionized water is used [12,17]. Finally, the magnetic plate is turned on, which drives a magnetic stirrer at 5 rpm on top of the frit, stirring the liquid-sample mixture (Fig. 3C). The flotation process is carried out at room temperature.

During the procedure, feldspar attaches to the bubbles due to its hydrophobic characteristics. The bubbles separate the feldspar from the quartz by carrying the grains to the upper part of the cell. Upon bursting of the bubbles at the liquid-air interface, the feldspars are released and drop into the flexible plastic tube attached to the upper part of the cell. The quartz particles, with their hydrophilic characteristics, remain at the bottom of the flotation cell (Fig. 3C). The flotation of a 90 g sample takes ≤2 h; less time is required if the sample contains less feldspar. After the flotation procedure, the quartz is filtered from the solution by rinsing with HCl (5%), washing with deionized water, and drying at <60 °C. The feldspar fraction is treated similarly and can be used for the analysis of other nuclides such as ^36^Cl, ^10^Be [30] or noble gases. The quartz extract is now ready for further CN chemistry, including the treatment with HF for removing atmospheric ^10^Be [7], and chemical extraction and separation of ^10^Be and ^26^Al [31].

## X-ray diffraction tracing of the quartz-enrichment process

The minerals separated from seven samples were investigated by XRD at the Helmholtz Institute Freiberg for Resource Technology (Table 1), using a PANalytical Empyrean device. It is equipped with a Co tube (1.7890 Å, operated at 35 mA and 35 kV), a Fe-beta-filter, and an X’Celerator Scientific area detector. Prior to the measurements, we milled ∼2 g sample aliquot to <0.5 mm grain-size for 8 min in ethanol in a McCrone mill with zirconia grinding elements. The samples were then dried at room temperature and homogenized. We transferred the sample aliquots into 24 or 16 mm diameter sample holder, using the back loading preparation method to minimize orientation effects. The irradiated area of the samples was held constant at 15 × 10 mm^2^, and, in the case of a smaller sample holder, used when the mineral yield was low, at 10 × 10 mm^2^. The samples were measured from 5 to 80° 2Θ with a step-width of 0.016° 2Θ and a total measurement time of 124 min. The data were evaluated using the Rietveld software bundle BGMN/Profex 3.3.0 [32]. For mineral composition analysis, we used aliquots from the following purification steps: (1) original sieved sample, (2) after magnetic separation, (3) after chemical cleaning, (4) and after flotation (Table 1).

**Fig. 4:**
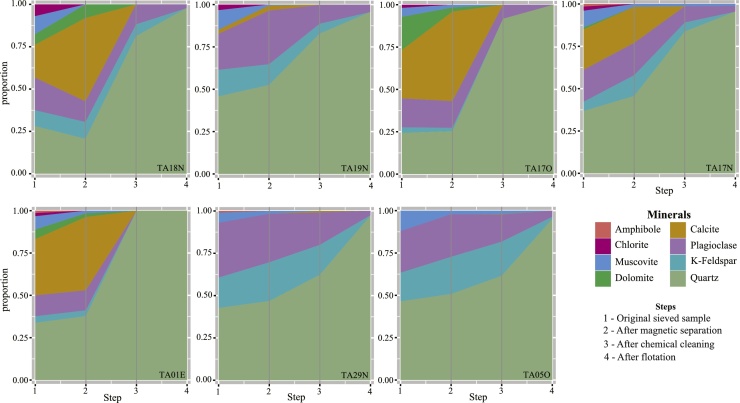
Mineral composition changes during the four-step quartz-enrichment procedure.

**Fig. 5:**
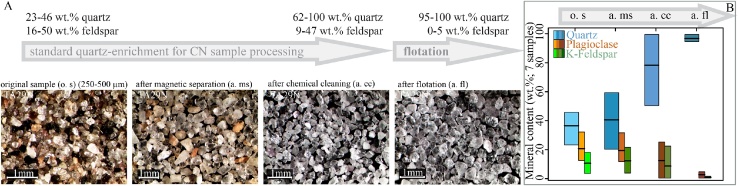
**(A)** Visual illustration of sample TA29N in its original stage and after each step of quartz enrichment. **(B)** Box plot showing the change in quartz, plagioclase, and K-feldspar content (in wt.%) after each quartz-enrichment step. Each box is the mean of the seven samples analysed.

Table 1 lists the location of the samples and their mineralogical compositions based on the XRD analysis. Fig. 4 illustrates the mineral composition in wt.% of the seven samples after each purification step. The entire clean-up procedure is summarized in Fig. 5(A). Fig. 5(B) highlights the step-wise quartz-enrichment, and the feldspar reduction. The main enrichment occurred after the six-cycle chemical cleaning and, in particular, the froth flotation. The XRD results show that in the original sieved sample material minerals other than quartz dominate. Three samples (TA19 N, TA29N, TA05O) contain only ∼45 wt.% quartz; the main other minerals are carbonates (dolomite, calcite), phyllosilicates (mica, chlorite), and feldspars. After magnetic separation, the quartz content increased only for two samples (TA19N, TA05O; Table 1). After the third purification step, the H/H_2_SiF_6_ treatment, the amount of quartz increased by at least 5 wt.%, leading to a minimum quartz content of ∼50.5 wt.%. The samples still contain feldspar in a range of 14.8 (TA17O) to 47.3 wt.% (TA05O), and also mica is still present in two samples (TA17N, 2.4 wt%; TA05O, 2.2 wt%). After the fourth and final step (flotation), quartz was enriched to at least 95 wt.% (TA05O); all other samples contained >97 wt.% of quartz. Mica was fully removed by flotation in one sample (TA05O), and partially in another (TA17N; Table 1).

## Discussion

The objective of the study was to obtain pure quartz from fluvial sediments for the extraction of CNs (^10^Be, ^26^Al). Pure quartz decreases contamination by meteoric ^10^Be and the content of Al. The analyzed samples, typical sand samples from riverbanks and terraces, contain high amounts of feldspar (Pl and K-fsp). In particular, Pl contains high amounts of Al. When quartz enrichment is insufficient, the contaminating minerals might impair the quantitative Be and Al separation during ion-exchange chemistry for the extraction of Be and Al. The contamination of the prepared “BeO” AMS target by Al_2_O_3_ or TiO_2_ from e.g. feldspar and mica results in less counts of ^10^Be with higher uncertainties in the ^10^Be statistics in the AMS analysis. The contamination with ^27^Al does not only affect the ^10^Be measurements but primarily those of ^26^Al, because when mineral phases other than quartz contribute high ^27^Al amounts, the ^26^Al/^27^Al ratio is lower and the statistical uncertainties are higher. Furthermore, the sea-level and high-latitude production rates are calibrated for pure SiO_2_; production-rate calculations must be done on similarly pure quartz samples [7,33].

This paper outlines an improved quartz purification device and procedure. We achieved high levels of quartz enrichment with a newly built flotation cell that uses a minimum of chemical reagents and allows processing of up to 90 g of sand samples in ≤2 h. Two samples yielded pure quartz concentrates, the remaining samples - very feldspar-rich initially - still contained up to 4.9 wt.% feldspar. After the froth flotation, these small amounts of feldspar will be dissolved during further chemical treatment, e.g., the first leaching cycle for the removal of atmospheric ^10^Be by concentrated HF.

Our cell has been successfully applied to obtain pure quartz and feldspar used in CN and OSL dating [20,23]. Samples of up to 90 g allow for good mixing by the magnetic stirrer and the froth flotation proceeds at a constant rate. With more than 90 g, the mixing becomes incomplete, and the flotation process might even stop due to overloading of the magnetic stirrer and difficulties in bubble generation. The usage of our flotation cell is versatile, as the concentration of the chemical reagents as well as the flux of bubbles is easily adjustable. In addition, the flotation procedure allows reducing the acid-wash chemical cycles (HCl/H_2_SiF_6_). A drawback of the flotation cell is its glass construction, which requires attention during use and cleaning due the danger of breakage. In addition, the use of HF, even at a 0.2% concentration, implies some risks. However, flotation procedures that use CO_2_ apply 1% HF, which is even more dangerous [18,19]. We stress that the flotation by-product, i.e., feldspar, although not described in this study, can be used for ^36^Cl, ^10^Be and noble gasses dating.

## Conclusions

We achieved separation of quartz from feldspar with a newly built flotation cell for our feldspar-rich samples from the Pamir, Central Asia. Introducing flotation to the sample preparation procedure ensures quartz enrichment and mitigates problems related to contaminations with other mineral phases in ^10^Be and ^26^Al cosmogenic nuclide dating. Our flotation cell is built of borosilicate glass, is designed for up to 90 g of sample material with grain sizes between 250–1000 μm, and achieves quartz and feldspar separation in ≤2 h. The operation procedure uses air bubbles to which the feldspars attach, 0.2% HF to reduce the surface energy of quartz, dodecylamine solution as a feldspar collector, and operates at a pH range of 2.4–2.7 at room temperature. In our samples, the quartz content increased from 23 to 46 wt.% in the untreated sample to 95–100 wt.% in the purified samples. The separated feldspar can be used for other CN applications, like ^36^Cl, ^10^Be, or noble gases. Our flotation method was also applied successfully for the separation of quartz and feldspar for other geochronologic methods, e.g., OSL dating. We recommend the use of flotation after physical quartz enrichment and after a single-step, acid-wash chemical cleaning cycle, reducing the chemical waste amount in comparison to the usually employed multiple cycles. The increased purity of quartz extract improves the quality of the subsequent steps of chemical sample treatment and consequently, of measurement results. Our results highlight flotation as a highly efficient step in CN sample preparation, which also has the potential of reducing costs for labor and reagents, and time.

## References

[1] Norris T.L., Gancarz A., Rokop D., Thomas K. (1983). Half-life of ^26^Al. J. Geophys. Res..

[2] Korschinek G., Bergmaier A., Faestermann T., Gerstmann U.C., Knie K., Rugel G., Wallner A., Dillmann I., Dollinger G., Lierse von Gostomski Ch., Kossert K., Maiti M., Poutivtsev M., Remmert A. (2010). A new value for the half-life of ^10^Be by Heavy-Ion Elastic Recoil Detection and liquid scintillation counting. Nucl. Instrum. Methods Phys. Res. Sect. B: Beam Interact. Mater. Atoms.

[3] Chmeleff J., von Blanckenburg F., Kossert K., Jakob D. (2010). Determination of the ^10^Be half-life by multicollector ICP-MS and liquid scintillation counting. Nucl. Instrum. Methods Phys. Res. Sect. B: Beam Interact. Mater. Atoms.

[4] Gosse J.C., Phillips F.M. (2001). Terrestrial in situ cosmogenic nuclides: theory and application. Quat. Sci. Rev..

[5] Muzikar P., Elmore D., Granger D.E. (2003). Accelerator mass spectrometry in geologic research. Bull. Geol. Soc. Am..

[6] Litherland A.E., Zhao X., Kieser W.E. (2011). Mass spectrometry with accelerators. Mass Spectr. Rev..

[7] Brown E.T., Edmond J.M., Raisbeck G.M., Yiou F., Kurz M.D., Brook E.J. (1991). Examination of surface exposure ages of Antarctic moraines using in situ produced ^10^Be and ^26^Al. Geochim. Cosmochim. Acta.

[8] Burtman V.S., Molnar P. (1993). Geological and geophysical evidence for deep subduction of continental crust beneath the Pamir. GSA Special Paper.

[9] Stübner K., Ratschbacher L., Rutte D., Stanek K., Minaev V., Wiesinger M., Gloaguen R., Project TIPAGE members (2013). The giant Shakhdara migmatitic gneiss dome, Pamir, India-Asia collision zone, I. Geometry and kinematics. Tectonics.

[10] Rutte D., Ratschbacher L., Schneider S., Stübner K., Stearns M.A., Gulzar M.A., Hacker B.R. (2017). Building the Pamir-Tibetan Plateau—crustal stacking, extensional collapse, and lateral extrusion in the Central Pamir: 1. Geometry and kinematics. Tectonics.

[11] Hacker B.R., Ratschbacher L., Rutte D., Stearns M.A., Malz N., Stübner K., Kylander-Clark A., Pfänder J.A., Everson A. (2017). Building the Pamir-Tibet Plateau—crustal stacking, extensional collapse, and lateral extrusion in the Pamir: 3. Thermobarometry and petrochronology of deep Asian crust. Tectonics.

[12] Herber L.J. (1969). Separation of feldspar from quartz by flotation. Am. Mineral..

[13] Vidyadhar A., Hanumantha Rao K., Forssberg K.S.E. (2002). Adsorption of N-tallow 1,3-propanediamine-dioleate collector on albite and quartz minerals, and selective flotation of albite from greek stefania feldspar ore. J. Colloid Interface Sci..

[14] Deer W.A., Howie R.A., Zussman J. (1992). An Introduction to the Rock Forming Minerals.

[15] Bulatovic S.M. (2015). Beneficiation of feldspar ore. Handbook of Flotation Reagents: Chemmistry, Theory and Practice.

[16] Fuerstenau D.W., Metzger P.H., Seele G.D. (1957). How to use this modified hallimond tube for better flotation testing. Eng. Min. J..

[17] Arnold R., Brownbill E., Ihle S. (1978). Hallimond tube flotation of scheelite and calcite with amines. Int. J. Miner. Process..

[18] Clifton T., Granger D.E., Gilbert Z., Caffee M. (2005). Quartz sample preparation for AMS. The 10th International Conference on AMS.

[19] Gibbon R.J., Granger D.E., Kuman K., Partridge T.C. (2009). Early Acheulean technology in the Rietputs Formation, South Africa, dated with cosmogenic nuclides. J. Hum. Evol..

[20] Fuchs M.C., Gloaguen R., Krbetschek M., Szulc A. (2014). Rates of river incision across the main tectonic units of the Pamir identified using optically stimulated luminescence dating of fluvial terraces. Geomorphology.

[21] Brookfield M.E. (2008). Evolution of the great river systems of southern Asia during the Cenozoic India-Asia collision: rivers draining north from the Pamir syntaxis. Geomorphology.

[22] Schwab M., Ratschbacher L., Siebel W., McWilliams M., Minaev V., Lutkov V., Chen F., Stanek K., Nelson B., Frisch W., Wooden J.L. (2004). Assembly of the Pamirs: age and origin of magmatic belts from the southern Tien Shan to the southern Pamirs and their relation to Tibet. Tectonics.

[23] Fuchs M.C., Gloaguen R., Merchel S., Pohl E., Sulaymonova V.A., Andermann C., Rugel G. (2015). Denudation rates across the Pamir based on ^10^Be concentrations in fluvial sediments: dominance of topographic over climatic factors. Earth Surf. Dyn..

[24] Schmidt J., Hacker B.R., Ratschbacher L., Stübner K., Stearns M., Kylander-Clark A., Cottle J.M., Alexander A., Webb G., Gehrels G., Minaev V. (2011). Cenozoic deep crust in the Pamir. Earth Planet. Sci. Lett..

[25] Stübner K., Ratschbacher L., Weise C., Chow J., Hofmann J., Khan J., Rutte D., Sperner B., Pfänder J.a., Hacker B.R., Dunkl I., Tichomirowa M., Stearns M.A. (2013). The giant Shakhdara migmatitic gneiss dome, Pamir, India-Asia collision zone: 2. Timing of dome formation. Tectonics.

[26] Schön J. (1983). Petrophysik. Physikalische Eigeschaften von Gesteinen und Mineralen.

[27] van der Plas L. (1966). Identification of Detrital Feldspar, Developments in Sedimentology.

[28] Altun N.E., Hicyilmaz C., Hwang J.-Y., Bagci A.S. (2006). Evaluation of a Turkish low quality oil shale by flotation as a clean energy source: material characterization and determination of flotation behavior. Fuel Process. Technol..

[29] Fuerstenau M.C., Jameson G.J., Yoon R.-H. (2007). Froth Flotation: A Century of Innovation.

[30] Zerathe S., Blard P.H., Braucher R., Bourlès D., Audin L., Carcaillet J., Delgado F., Aumaître G., Keddadouche K., Benavente C. (2017). Toward the feldspar alternative for cosmogenic ^10^Be applications. Quat. Geochronol..

[31] Merchel S., Herpers U. (1999). An Update of Radiochemichal Separation Techniques for the Determination of Long-Lived Radionuclides via Accelerator Mass Spectrometry. Radiochim. Acta.

[32] Doebelin N., Kleeberg R. (2015). Profex: a graphical user interface for the Rietveld refinement program BGMN. J. Appl. Crystallogr..

[33] Merchel S., Gärtner A., Bookhagen B., Chabilan A., Gurlit S. (2018). Attempts to understand potential deficiencies in chemical procedures for AMS: Cleaning and dissolving quartz. Nucl. Instr. Meth. Phys. Res. B.

